# Characterization and stability evaluation of Egyptian propolis extract nano-capsules and their application

**DOI:** 10.1038/s41598-023-42025-0

**Published:** 2023-09-26

**Authors:** Azza A. Amin, Khaled F. Mahmoud, Manal F. Salama, Vincenzo Longo, Luisa Pozzo, Effat I. Seliem, Mona A. Ibrahim

**Affiliations:** 1 Department of Food Technology, National Research Centre (NRC), Dokki, 12622 Egypt; 2 Institute of Agricultural Biology and Biotechnology (IBBA), National Research Council (IBBA-CNR), Via Moruzzi 1, 56124 Pisa, Italy

**Keywords:** Nanobiotechnology, Structure determination, Nanobiotechnology, Other nanotechnology

## Abstract

The increasing demand for natural products and biotechnological activities from bees facilitate their widespread use in food preservation and beneficial effects on humans. This study aimed to prepare and characterize the nano-capsules of Qaluiobia (PQG) governorates propolis extracted with water, ethanol and supercritical fluid-carbon dioxide at 50 °C with co-solvent. Propolis bioavailability was analyzed and introduced to prepare crackers to extend their shelf life. Nano-encapsulation was examined using transmission electron microscopy (TEM), differential scanning calorimetry (DSC) and antioxidant activity. Ethanol and supercritical fluid-carbon dioxide (SCF-CO_2_) at 50 °C with ethanol as co-solvent recorded higher yield, antioxidant activities, total phenolics and total flavonoids. SCF-CO_2_ extracts had a higher flavonoid concentration. It was revealed that propolis nano-capsules had high-temperature stability and cytotoxic effects against the three tested human cancer cell lines (i.e. PC3, MCF7 and HePG2). The higher overall acceptability of crackers fortified with PQG was achieved with SCF-CO_2_ at 50 °C and ethanol extract nano-capsules, i.e. 86.57% and 86.29% respectively. The higher ability to retain antioxidant activity reduces the increase of peroxide value (PV), preventing rancidity and increasing the shelf life of crackers during the storage period. Practical application: This study can provide a suitable method for extracting bioactive compounds from propolis, and improve the biological properties and activities by nano-encapsulation, also reveals the extent of its use as a natural antioxidant and anticancer and its application in bakery products as a functional food.

## Introduction

Propolis is a complex resinous produced by honey bees and has highly different physical characteristics depending mainly on the environmental conditions^1^. It is well known that the biochemical compounds in propolis contain phenolic compounds, vitamins, carbohydrates, hydrocarbons and carboxylic acids, mainly responsible for therapeutic effects and high antioxidant activity^2,3^.

Raw propolis is not soluble in water and is extracted by solvents. Propolis is purified to preserve its polyphenolic fraction. These polyphenolic compounds are essential for healing effects^4^. The ingredients extracted from propolis exhibit many biological activities and health benefits and can be used as functional ingredients in food and medicine^4^.

Propolis is complex and contains 30% wax, 50% resin (mainly flavonoids and phenolic acid derivatives), 10% aromatic oils, 5% other organic residues and 5% pollen^5^. The chemical composition of propolis, especially related to its components and their polarity, makes it challenging to use in a free or non-encapsulated form in various applications. The extraction yield of bioactive components depends on the extraction methods and organic solvents (i.e. methanol, chloroform, ethanol and ethyl-ether). Many researchers have studied propolis processing using conventional methods^6,7^. However, despite its use, it has some shortcomings, mainly due to the quality of processing resulting in the presence of solvent residues in the final product.

Supercritical fluid (SCF) extraction is an innovative technology that has gained widespread interest due to increasingly restrictive environmental regulations (i.e. the use of low temperatures, reduced solvent use and energy consumption)^8–10^. This technology has spread in multiple industrial areas due to its unique advantages and SCF characteristics^11,12^. EPE has the potential to be used as a natural additive with antimicrobial and antioxidant characteristics in toast bread^13^. Still, SCF is a promising alternative technique in the fine chemistry, foodstuff fields and pharmaceuticals^14^.

Nanotechnology is an innovative technique for various applications in nano-food and nano-medicine. Nano-particles with encapsulation produce small size, large surface area and protection, with high bioavailability compared to their bulk counterparts^15^.

Nano-capsulation can be obtained using emulsification, high-pressure homogenization, evaporation, spray drying, ultrasonication, high-speed stirring, micro-emulsion and ball milling^16^.

This work evaluated Qaluibia (PQG) propolis extraction produced methods using extraction methods (i.e. water, ethanol 95% and one green technology SCF-CO_2_ at 50 °C) as the extractants were used. The physicochemical properties, nano-capsulation, cytotoxic activity of propolis and their application as functional bakery products were also studied.

## Materials and methods

### Materials and chemicals

Egyptian raw honey bee’s propolis was collected from Delta region Qaluiobia Governorate (PQG) and kept in dark sterile glass containers at room temperature until further use. All chemicals and reagents i.e. Folin–Ciocalteu reagent, 2,2-diphenyl-1-picryl hydrazyl radical (DPPH), Tween 20 (T_20_), Ethanol, Aluminum chloride, Potassium acetate, linoleic acid, Gallic acid, Sodium alginate, Butylated hydroxyltoluene (BHT), Sodium Thiocyanate, Ascorbic acid, Quercetin, Methanol, Phosphate buffer, Ammonium thiocyanate, Ferrous chloride, Potassium ferricyanide, Trichloroacetic acid, Ferric chloride, Sodium carbonate, Aluminum chloride, Formic acid, Acetonitrile, Phosphotungestic acid, Chloroform, Glacial acetic acid, Sodium thiosulphate were of analytical grade and purchased from Sigma Aldrich Chemical (Merck KGaA, Darmstadt, Germany).

The work does not involve humans or conduct experiments on live animals.

### Methods

#### Extraction methods

##### Propolis water extracts (PWE)

Honey bees propolis powders (10 g) were suspended in 100 ml distilled water and shaken for 24 h at 24 °C. The suspension was then centrifuged at 3000 rpm for 20 min at 4 °C, and the supernatants were collected after several extractions (3 times) under the same conditions, dialyzed against distilled water, and lyophilized (LABCONCO, Freeze dryer, Console, 12 L −50 °C, 240 V, Catalog No. 7754030, USA)^17^. The yield of water extract was defined as the mass of water extract of propolis obtained by dry mass of raw propolis used in percentage.

##### Propolis ethanolic extracts (PEE)

Ethanolic extracts of propolis (PEE) were obtained using the method of Yong and Masaharu^18^ with some modification where, (10 g) propolis powder was mixed with 250 methanol (95%) at room temperature in a dark place for 24 h. The mixture was centrifuged (3000 rpm, for 10 min) to obtain the supernatant. The propolis yield was calculated based on the initial amount of dry propolis in percentage.

##### Propolis supercritical fluid carbon dioxide extracts (PSCF-CO_2_)

The extracts of propolis were carried out at temperatures of 50 °C and 250 bar pressure using a laboratory-scale unit in National Research Centre (Speed TM SFE-2/4, Applied separations, Built in conjunction with the USDA1- USA). Ten grams of propolis powder were mixed with ethanol 15% (w/w) as co-solvent. The CO_2_ was pumped into the reactor, which was supported by two: 300 vessels and kept for 30 min to allow complete contact and guarantee that the operational conditions of temperature and pressure were stabilized. The CO_2_ mass flow rate was 1.0 g/min. The samples were collected, and the process line was washed with the used ethanol to recover the extract deposited. The global extract yield Xo (%) (extraction + cleaning process) to the initial mass of raw material (drybases)^19^.

At the end of the extraction methods, the yield of propolis was collected and calculated as follows:

1 $${\text{Yield }}\left( \% \right) \, = \, \left( {{\text{W}}_{{1}} /{\text{W}}_{{2}} } \right) \, \times {1}00$$

where: W _1_ is the weight of propolis extracts (g) and W_2_ is the weight of dried raw propolis powder (g).

#### Preparation of nano-capsules

Ethanol and SCF-CO_2_ Propolis extracts were encapsulated into nano-forms using ultrasound and high-speed homogeniser (PRO, USA) methods, according to Vasiliki and Constantina^20^. Nano-emulsion was prepared by adding one gram propolis extract to 10 ml deionized water (water (1:1) with addition of 0.1% T_20_. The water was gradually added with stirring using a magnetic stirrer (2000 rpm at room temperature) to avoid the formulation of bubbles during mixing till complete dissolving, then homogenized using high-speed homogenizer (Model: 400ELPC, PRO Scientific Inc., 01-02411ELPC HOMOGENIZER, USA) at 18,000 rpm for 30 min in the presence of an ice water bath to reduce the temperature of the mixture. The sample was stored at 4 °C for 24 h before encapsulation. For encapsulation, 50 ml sodium alginate solution (prepared by adding 3 g sodium alginate to 100 ml deionized water and mixed by magnetic stirrer at 2000 rpm for 60 min and left in a refrigerator at 4 °C to form a gel) were gradually added to 10 ml nano extract emulsion (5:1) using homogenizer (2000 rpm, 10 min) and treated by ultrasound (Sonics & Materials Inc., Newton, Connecticut, USA) for 30 min at 30 °C then the encapsulated emulsion was packed in brown packages at 4 °C till use.

#### Chemical properties of propolis extracts and their nano-capsules

##### Total phenolic content (TPC)

The total phenolic content (TPC) of three extracts and their nano-capsules were determined spectrophotometrically using Folin-Ciocalteu reagent according to the method described by Ebrahimzadeh et al*.*^21^ with some modification. The extracted samples and their nano-capsules (0.5 ml) were mixed separately with Folin-Ciocalteu reagent (5 ml with distilled water at a rate of 1:10) for 3 min then; 3 ml of 2% sodium carbonate (1 M) was added. The mixture was left for 15 min, and the polyphenols were determined by an automated UV–VIS spectrophotometer at 765 nm and the results were calculated using a Gallic acid calibration curve (0–100 mg/l). The blank was prepared using the same procedure with 0.5 ml of pure water in place of the extract. The results are expressed as equivalents to Gallic acid (mg GAE/g dry extract).

##### Total flavonoid content (TFC)

The total flavonoid content (TFC) of three extracts and their nano-capsules were determined according to the method described by Huang et al*.*^22^, and Ebrahimzadeh et al*.*^21^, Nabavi et al*.*^23^. The propolis extracts and their nano-capsules (0.5 ml) were mixed separately with 1.5 ml methanol, 0.1 ml of 10% aluminium chloride, 0.1 ml of 1 M potassium acetate and 2.8 ml of distilled water. They then left at room temperature for 10 min. The absorbance of the mixture was measured at 415 nm on a UV/visible spectrophotometer. The quercetin (µg/g) was used as a standard for the calibration curve.

##### Determination of radical-scavenging activity by (DPPH)

Radical scavenging activity of tested extracts ability was assayed using the method of Hatano et al.^24^. Different concentrations of three extracts (i.e.10, 20, 30, 40, 50 and 60 µg/ml) were added to reaction solution DPPH (1 ml) (0.2 mM). The mixture was shaken forcibly and left at room temperature for 30 min, and then the absorbance of the solution was measured spectrophotometrically at 517 nm.

2 $$\% {\text{ DPPH \, radical \, scavenging \,activity }} = \, \left( {\left( {{\text{Ac}} - {\text{As}}} \right)/{\text{Ac}}} \right) \, \times {1}00$$

As is the absorbance of the sample; Ac is the absorbance of control in the absence of the sample.

Nano-capsules of ethanol and SCF-CO_2_ with (60 µg/ml) were determined as above.

##### Reducing power (RP)

The reducing power (RP) of three extracts were determined following the method of Oyaizu^25^. Briefly, different concentrations of extracts (10, 20, 30, 40, 50 and 60 µg/ml) were mixed with 5 ml phosphate buffer (0.2 M, pH 6.6) and 2.5 ml potassium ferricyanide (1%) and compared with the same concentration of BHT and ascorbic acid in ethanol (95%) with 2.5 ml of sodium phosphate buffer (200 mM, pH 6.6). The reaction mixture was incubated at 50 °C for 20 min. Aliquots of 1% trichloroacetic acid (5 ml) were added to the mixtures, then centrifuged at 2000 rpm for 10 min, and the absorbance of the pink color mixture was recorded spectrophotometrically at 700 nm.

Nano-capsules of ethanol and SCF-CO_2_ with (60 µg /ml) were determined as above.

##### Total antioxidant activity (TAA)

Water, Ethanol and CO_2_ extracts total antioxidant activity (TAA) were determined using a linoleic acid system^26^. The reaction mixture at different extract concentrations (i.e.10, 20, 30, 40, 50 and 60 µg/ml) were separately treated with linoleic acid (0.13 ml), phosphate buffer 0.2 M (pH 7.0, 10 ml) and ethanol (99.8%). The mixture was adjusted to 25 ml by distilled water. Then incubated at 40 °C for 10 min and the oxidation rate was measured using thiocyanate method^27^. The obtained solutions were added to a mixture of 10 ml ethanol (75%) with 0.2 ml ammonium thiocyanate (30%), 0.2 ml of sample solution and 0.2 ml of ferrous chloride solution (20 mM in 3.5% HCl), stirring for 3 min and the peroxide value was measured using a spectrophotometer (JASCO, Corporation Model V-730, S.N. A112961798, Tokyo, Japan) at 500 nm. The percent inhibition of linoleic peroxidation is calculated as 100 – (Abs increase of sample/Abs increase of control X100). The commercial antioxidants butylated hydroxytoluene (BHT) and ascorbic acid were used.

Nano-capsules of ethanol and SCF-CO_2_ with (60 µg/ml) were determined as above.

#### Physical properties of nano-capsules

The nano-capsules formed were tested by their efficiency, yield, TEM, DSC effect as follows:

##### Encapsulation efficiency (EE)

Encapsulation efficiency (% encapsulation) was calculated following Busch et al*.*^28^ based on the total amount of phenolic in encapsulated propolis per total amount of phenolic in propolis extract. Encapsulation efficiency can be calculated by the following formula:

3 $$\% {\text{Encapsulation}} = x/y \, \times \, 100$$

where *x* is the total amount of phenol in encapsulated propolis (%); *Y* is the total amount of phenol in propolis extract (%).

##### Encapsulation yield (EY)

The encapsulation yield (EY) is another factor to be considered during an encapsulation process. The effectiveness of encapsulation yield (EY) was measured using Paviani et al*.*^19^ method and calculated as follows:

4 $${\text{EY }}\left( \% \right) \, = \frac{{\text{Total \, mass \, of \, the \, sample \,after \, encapsulation}}}{{\text{Total \, mass \, of \,the \,sample \, before \, encapsulation}}} \,\times \,{1}00$$

##### Transmission electron microscopy (TEM)

Propolis extracts and their nano-capsules' morphological characteristics were tested by TEM (1400, JEOL, Japan) using Saloka et al*.*^29^ method. The nano-particles suspensions were dripped onto a 400-mesh copper grid coated with Forvar and stained by 2% phosphotungstic acid^30^. The samples were air-dried at room temperature for more than 2 h before analyzing on the TEM.

##### Thermal stability (DSC)

Thermal stability of propolis extract and their nano- capsules were measured using a differential scanning calorimeter (DSC) (Mettler Toledo, SWITZERLAND) according to Hazra et al*.*^31^ as follows. Ten milligrams of both samples were placed in aluminium crucibles under a flow of nitrogen gas (40 ml/min). A dynamic scan was performed at a heating rate of 10 °C/min over a temperature range of −150 to 300 °C. Evaporation enthalpies were calculated by peak area integration of DSC profiles, and the results were compared with the estimated vaporization enthalpy of major components.

##### LC–ESI–MS/MS analysis of propolis forms

The analysis of crude propolis, nano-capsules propolis extracts by slovent and SCF-CO_2_ at 50 °C was performed using liquid chromatography–electro-spray ionization–tandem mass spectrometry (LC–ESI–MS/MS) with an Exion LC/AC system for separation and SCIEX Triple Quad 5500+ MS/MS system equipped with an electro-spray ionization (ESI) for detection. The instrument data were collected and processed using the SCIEX OS 1.6.10.40973 software.

The separation of the targeted analyses was performed with a Discovery^®^ BIO Wide Pore C18-5 Column (4.6 × 250 mm, 5 µm). The mobile phases were consisted of two eluents A: 0.1% formic acid in water; B: 0.1% formic acid in acetonitrile (LC grade). The mobile phase gradient was programmed as follows: 5% B at 0 min, 5–25% B from 0.0 to 60.0 min, 25–5% B from 60 to 65 min, 5% from 65 to 70. The flow rate was 1.0 ml/min and the injection volume was 5 µl. For MS/MS analysis, negative ionization mode was applied with a scan (EMS-IDA-EPI) from 150 to 800 Da with the following parameters: curtain gas: 25 psi; ion spray voltage: −4500; source temperature: 400 °C; ion source gas 1 & 2 were 55 psi.

##### Cytotoxic effect on human cell lines

Cell viability was assessed by the mitochondrial-dependent reduction of yellow MTT [3-(4,5-dimethylthiazol-2-yl)-2,5-diphenyl tetrazolium bromide] to purple formazan^32^.

Cells from (National Cancer Institute, Egypt) were suspended in DMEM-F12 medium (for HePG2, MCF7, and PC3) beside one normal cell line (BJ1), 1% antibiotic–antimycotic mixture (10,000 U/ml Potassium Penicillin, 10,000 µg/ml Streptomycin Sulfate and 25 µg/ml Amphotericin B) and 1% l-glutamine at 37 °C under 5% CO_2_.

Cells were batch cultured for 10 days, then seeded at concentration of 10 × 10^3^ cells/well in fresh complete growth medium in 96-well microtiter plastic plates at 37 °C for 24 h under 5% CO_2_ using a water-jacketed CO_2_dioxide incubator (Sheldon, TC2323, Cornelius, OR, USA). Media was aspirated, fresh medium (without serum) was added, and cells were incubated either alone (negative control) or with different concentrations of sample to give a final concentration of (100–50–25–12.5–6.25–3.125–0.78 and 1.56 µg/mL). After 48 h of incubation, the medium was aspirated, 40 µL MTT salt (2.5 µg/mL) were added to each well and incubated for a further 4 h at 37 °C under 5% CO_2_. To stop the reaction and dissolve the formed crystals, 200 µL of 10% Sodium dodecyl sulfate (SDS) in deionized water were added to each well and incubated overnight at 37 °C.

A positive control which composed of 100 µg/ml was used as a known cytotoxic natural agent that gives 100% lethality under the same conditions^33^. The absorbance was then measured using a microplate multi-well reader (Bio-Rad Laboratories Inc., model 3350, Hercules, California, USA) at 595 nm and a reference wavelength of 620 nm. The percentage of change in viability was calculated according to the formula:

5 $$[\left( {{\text{Absorbance \,of \,extract }}/{\text{absorbance \,of \,negative \,control }} - {1}} \right)] \, \times { 1}00$$

Using the SPSS 11 program, the IC_50_ was determined using probit analysis. The degree of selectivity of the synthetic compounds was expressed as SI = IC_50_ of the pure compound in a normal cell line/IC_50_ of the same pure compound in a cancer cell line, where IC_50_ is the concentration required to kill 50 percent of a cell population.

#### Application

##### Crackers preparation

With slight modification, five types of crackers were prepared according to the method of Benjakula and Karnjanapratum^34^. The control crackers were prepared using 62.5% wheat flour (72%), 1.16% salt, 0.23% sugar, 0.37% baking powder, 15% sunflower oil, 0.187% paprika and 20.25% water to produce the dough. The ingredients were mixed at a low speed for 3 min. The resulting cracker dough was sheeted to a thickness of a 0.4 mm and cut into a rectangle (2.4 × 7.3 cm^2^). The shaped cracker dough was baked in an electric oven at 120 °C for 30 min (SL-Shel-LAB-1370FX). In the same way, the remaining cracker samples were prepared with the same previous ingredients to produce 1.8 g of four types of crackers containing 0.6 g of nano-capsules of PEE (Propolis ethanolic extract), PSCF-E (Propolis Supercritical Extract at 50 °C), PNC-EE (Propolis Nano-Capsule Ethanolic Extract) and PNC-SCFE (Propolis Nano-capsule Supercritical Extract at 50 °C) (1.8 g capsule contain 0.6 g propolis extract of their nano-forms).

##### Sensory evaluation of crackers

All samples of the crackers were sensory assessed by ten-member panels for appearance, color, thickness, texture, shrinkage, taste and odor using the method described by Smith^35^.

#### Storage stability of crackers

The stability of the stored cracker samples (at 25 °C for 90 days) was determined every month compared to the control sample using the DPPH and PV previous described methods.

##### Determination of radical-scavenging activity using (DPPH)

The oils from each cracker sample were extracted by n-hexane (25 ml) that was evaporated after keeping it in a refrigerator for 24 h using rotary evaporator^36^. The antioxidant activity of the crackers’ residue was determined as described previously.

##### Determination of peroxide value (PV)

Peroxide value of all cracker samples fortified with propolis extracts and their nano-forms were determined by extracting the oil from samples with n-hexane, and then PV was performed according to AOAC^36^. Two mg of oil were added and mixed in a solution containing chloroform-glacial acetic acid (30 ml, 3:2 v/v) and sodium thiosulphate (1 ml, 0.1 M) until the disappearance of yellow color. PV (meq/kg) was calculated as follows:

6 $${\text{PV }}\left( {{\text{meq}}/{\text{kg}}} \right) \, = {\text{ C}} \times \left( {{\text{V}} - {\text{V}}_{0} } \right) \times {12}.{69} \times {78}.{8}/{\text{m}}$$

where; C is sodium thiosulphate concentration (mol/l), V and VO volumes of sodium thiosulphate blank respectively (ml), and m is the mass of cracker sample extracts (mg).

#### Statistical analysis

Results were expressed as means ± standard deviation (n = 3) and ANOVA variance analysis with average comparison Duncan’s^37^ Multiple Range set to ˂0.05. All the statistical processes were conducted by the Statistical Package for Social Science (SPSS, V_21.0_) for Windows (SPSS, Inc., Chicago, IL, USA).

## Results and discussion

### Physical and chemical properties of propolis extracts

#### Efficiency of extraction yield methods

The yield of the different extraction methods was determined as tabulated in Table 1.

**Table 1:** Total yield of propolis samples extracted by traditional and green techniques.

Type of extraction	Techniques	Yield (g/100 g dw) of propolis
Water extract	Traditional extract	14.63 ± 0.56^b^
Ethanol extract	Traditional extract	19.21 ± 0.75^bc^
SCF-CO_2_ at 50 °C	Green extract	24.26 ± 0.82^cd^

*SCF-CO*_*2*_ super critical fluid-carbon dioxide.

Different superscripts in the same column or row are significant differences at P ≤ 0.05 level.

In the above Table, the propolis extraction yield differences were shown as affected by the three extraction methods. Significant differences among values (p ˂ 0.05) were found in the extracted yields of the brown propolis (PQG). Water extraction had a lower yield, due to the high polarity of water resulting from polarization of its molecule what makes this solvent suitable for extraction of lyphylic organic compounds present in propolis. Although the polarity of water was high, the yield was low because it was not the only factor affecting the extraction efficiency^38^. The yield of water extraction of PQG was 14.63 g/100 g dw and agreed with that obtained by Biscaia and Ferreira^39^, at 14.3%.

The highest yields obtained with SCF-CO_2_ with co-sovent at 50 °C and 250 bar for PQG was 24.26%. These high pressures increase the density and polarity of carbon dioxide which increase its capacity to extract more polar components^19^*.*

#### TPC and TFC of propolis extracts

The optimum phenolic content varied according to the plant types and active compounds^40^ in which Phenolic compounds can be classified into polar and weak-polar^41^. The most crucial feature of propolis extracts was polyphenols in their structure, which is an indication for high biological activity. The total contents of polyphenols and flavonoids in extracts of the propolis obtained using the three extraction methods are shown in Table 2.

**Table 2:** Total phenolic and flavonoids of propolis extracts.

Types of extraction	TPC(GA/G)	TFC((QE/G)
Water extract	162.33 ± 2.7^a^	366.51 ± 3.9^b^
Ethanol extract	318.36 ± 3.8^c^	653.73 ± 6.8^e^
SCF-CO_2_ extract at 50 ºC	395.22 ± 4.1^d^	745.42 ± 8.4^f^

*TPC* total polyphenolic content, *TFC* total flavonoids content, *SCF-CO*_*2*_ super critical fluid-carbon dioxide.

Different superscripts in the same column or row are significant differences at P ≤ 0.05 level.

Results showed that the highest TPC value for CO_2_ extract at 50 °C was 395.22 ± 4.1, followed by ethanolic sample 318.36 ± 3.8, while the water samples had the lowest activity.

When the total flavanoids amount (TFC) was examined, it was found that samples extracted with CO_2_ extractor at 50 °C had the highest flavonoid value being 745.42 ± 8. All supercritical extracts presented higher flavonoids concentration to the initial value found in three extracted method,). Flavonoid level in ethanol extract is lower than that extracted by CO_2_ as ethanol belongs to the group of less polar solvent. While, water extract showed lower value for TFC being 366.51 ± 3.9.

The total concentration of polyphenols or flavonoids was not the only factor responsible for antioxidant properties but also, the chemical nature of polyphenols and the presence of other compounds contributed to the overall antioxidant capacity of the extracts^42^.

#### Antioxidant activity of propolis extracts

##### DPPH, reducing power (RP), and total antioxidant activity (TAA)

**Figure 1:**
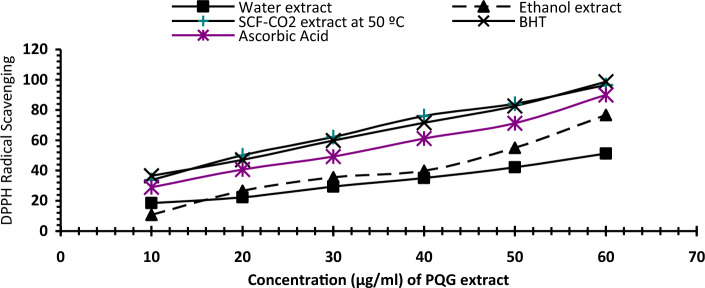
DPPH scavenging activities (%) of different extracts. BHT and ascorbic acid were used as positive controls. Where; *SCF-CO*_*2*_ super critical fluid-carbon dioxide.

**Figure 2:**
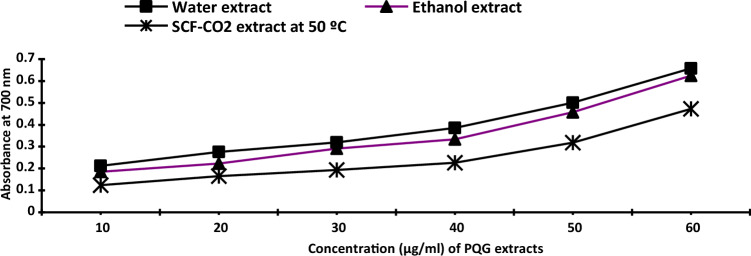
The reducing power of different extracts. BHT and ascorbic acid were used as positive controls. Where; *SCF-CO*_*2*_ super critical fluid-carbon dioxide.

**Figure 3:**
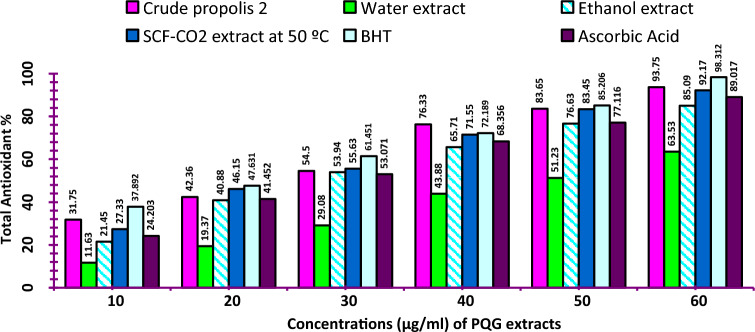
Total antioxidant activity (TAA %) of propolis extracts. BHT and ascorbic acid were used as positive controls. Where; *SCF-CO*_*2*_ super critical fluid-carbon dioxide.

The antioxidant properties determined using DPPH, reducing power methods and TAA of propolis extracts at different extracting methods in Figs. 1, 2 and 3 have been thoroughly investigated and compared. Propolis extracts were characterized by the high or similar values of scavenging activity, RP, and TAA when compared to the synthetic BHT and ascorbic acid in vitro studies. Propolis extracts DPPH scavenging activity ranged from about 51.16% to 96.54% using different extraction methods. PQG antioxidant activities in CO_2_ extract at 50 °C were higher than other extracts in Fig. 1.

Reducing power was related to antioxidant activity because antioxidants can give off their electrons to reduce reactive radicals in Fig. 2. Thus, the reducing power could indicate the antioxidative potential of prospective antioxidants. The increase in the absorbance values for the method of determining reducing power was due to the phytochemical components present.

At the same time, RP had a slight increase from 0.624 to 0.692 at ethanolic extract and from 0.472 to 0.484 (Abs. at 700 nm) for SCF-CO_2_.

SCF-CO_2_ extraction showed the highest value (92.17%) followed by ethanol being 85.09%. while; sample extracted by water had lower TAA content. These results were in agreement with those reported by Paviani et al*.*^19^. Generally, water extract showed lower values for TPC, TFC and TAA than the others extracted methods; therefore it was excluded from the nano procedure.

#### Antioxidant activity of Propolis nano-capsule bioactive compounds

Propolis nano-capsules had significant differences among values in Table 3 (p ˂ 0.05) and had the highest TPC and TFC values for CO2 extract at 50 °C equal to 381.86 GAE/g and736.92 mg PE/g, respectively. This was followed by the ethanolic sample (286.12 GAE/g and 561.58 mg PE/g, respectively.

**Table 3:** Antioxidant activities of propolis nano-capsules bioactive compounds.

Items	Antioxidant activity of propolis			
	Ethanol extract		SCF-CO_2_ extract at 50 °C	
	Free extract	Nano-capsules	Free extract	Nano-capsules
TPC (mgGA/G)	318.36 ± 3.8^a^	286.12 ± 3.2^ab^	395.22 ± 4.1^b^	381.86 ± 3.9^ab^
TFC (QE/G)	653.73 ± 6.8^c^	561.58 ± 7.1^b^	745.42 ± 8.4^d^	736.92 ± 8.0^cd^
RP (Abs)	0.624 ± 0.05^b^	0.692 ± 0.05^c^	0.472 ± 0.03^a^	0.484 ± 0.03^ab^
DPPH (%)	76.73 ± 2.8^b^	64.88 ± 2.6^a^	96.54 ± 3.1^c^	92.57 ± 2.9^c^
TAA (%)	85.092 ± 4.62^ab^	76.183 ± 4.31^a^	92.173 ± 5.02^c^	89.584 ± 4.81^b^

*TPC* total polyphenolic content, *TFC* total flavonoids content, *SCF-CO*_*2*_ super critical fluid-carbon dioxide, *RP* reducing power, *TAA* total antioxidant activity.

Different superscripts in the same column or row are significant differences at P ≤ 0.05 level.

The propolis extract obtained using ethanolic and supercritical fluid CO_2_ methods differ significantly between each other in terms of antioxidant activity (Table 3). Sodium alginate-propolis ethanolic extract nano-capsules significantly decreased by nearly 10%, 14%, 10, 15% and 10% for TPC, TFC, RP, DPPH and TAA, respectively than ethanolic free extracts. While, the percentage decrease between the SCF-CO_2_ extract and its nano-capsules was lower (3.5, 1, 2.5, 4 and 3%), respectively. The nano-capsules of SCF-CO_2_ extract maintained the stability of the bioactive compound despite the ethanolic extract in all antioxidants parameter.

Falcão et al*.*^43^ proved that European propolis samples (Russia and Italy) had similar polyphenol components and antioxidant activity of Brazilian propolis, which contained fewer polyphenols and fewer antioxidant properties.

In addition, SCF-CO_2_ presents good yields and nano-particles preserves the physico-chemical characteristics of the components to be extracted^14^, and improve the bioavailability of the polyphenolic compound^44^.

### Physical properties of nano-capsules

Nano-capsules formed were tested by their efficiency, yield, TEM, DSC, LC–ESI–MS/MS and cytotoxic effect as follows:

#### Encapsulation efficiency (%)

The encapsulation efficiency of Propolis ethanol and SCF-CO_2_ at 50 °C extracts was 84.56% and 95.89%, respectively. This was in agreement with Chen et al*.*^45^, who reported an adequate encapsulation efficiency of at least 80%.

#### Encapsulation yield (%)

The encapsulation yield of Propolis SCF-CO_2_ extract at 50 °C was significantly higher than that of the ethanolic extract. The nano-capsules of PQG-SCF-CO_2_ extract at 50 °C were 95.89% more effective than ethanol extract (84.56%) based on the basic structure of total flavonoid (736.92). The nano-capsules were also more hydrophobic than the total phenols (381.86). The possibility that sodium ALg bound the hydroxyl groups found in flavonoids with hydrogen bonds^46^ was supported by these data.

#### Transmission electron microscopy (TEM)

**Figure 4:**
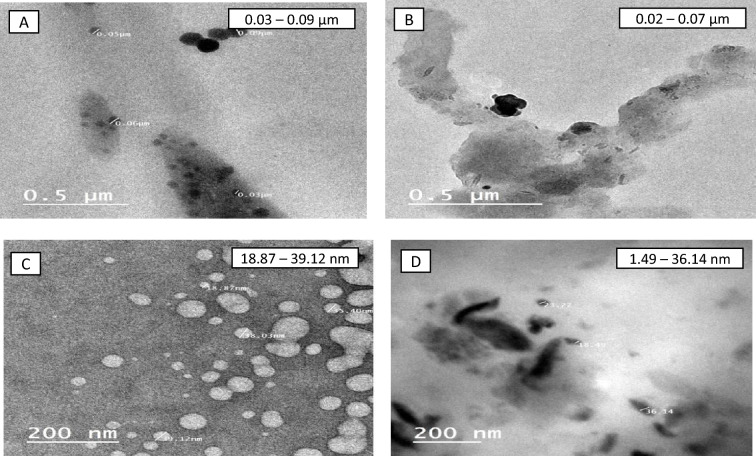
(**A–D**) TEM image of propolis extracts before and after nano-capsulation. (**A**) TEM of propolis ethanol extract; (**B**) TEM of propolis SCF-CO_2_ extract at 50 °C. (**C**) TEM of propolis ethanol extract nano-capsules; (**D**) TEM of propolis SCF-CO_2_ extract nano-capsules at 50 °C.

The morphology and particle size of the propolis nano ethanolic and SCF-CO_2_ at 50 °C extracts were measured using TEM. These results are shown in Figs. 4A, B. Propolis extracts had a large particle size in the micrometre range, with an average particle size between 0.03 and 0.09 μm for the ethanol extract and 0.02 to 0.07 μm for the SCF-CO_2_ extract at 50 °C. The propolis ethanol extract in sodium-ALg nano-particles was homogenous, had no aggregation, had a round shape and smooth surface and were discrete, with an average ranging from 18.87 to 39.12 nm, as shown using a TEM image in Fig. 4C. The SCF-CO_2_ extract at 50 °C had spherical shape, had no aggregation and homogenous, with an average ranging from 1.49 to 36.14 nm in Fig. 4D, compared to the encapsulated ethanol extract^47^. This result may be due to the anionic carboxylic groups of sodium-ALg, which caused strong electrostatic repulsion between the particles^48^.

#### Differential scanning calorimetry (DSC)

**Figure 5:**
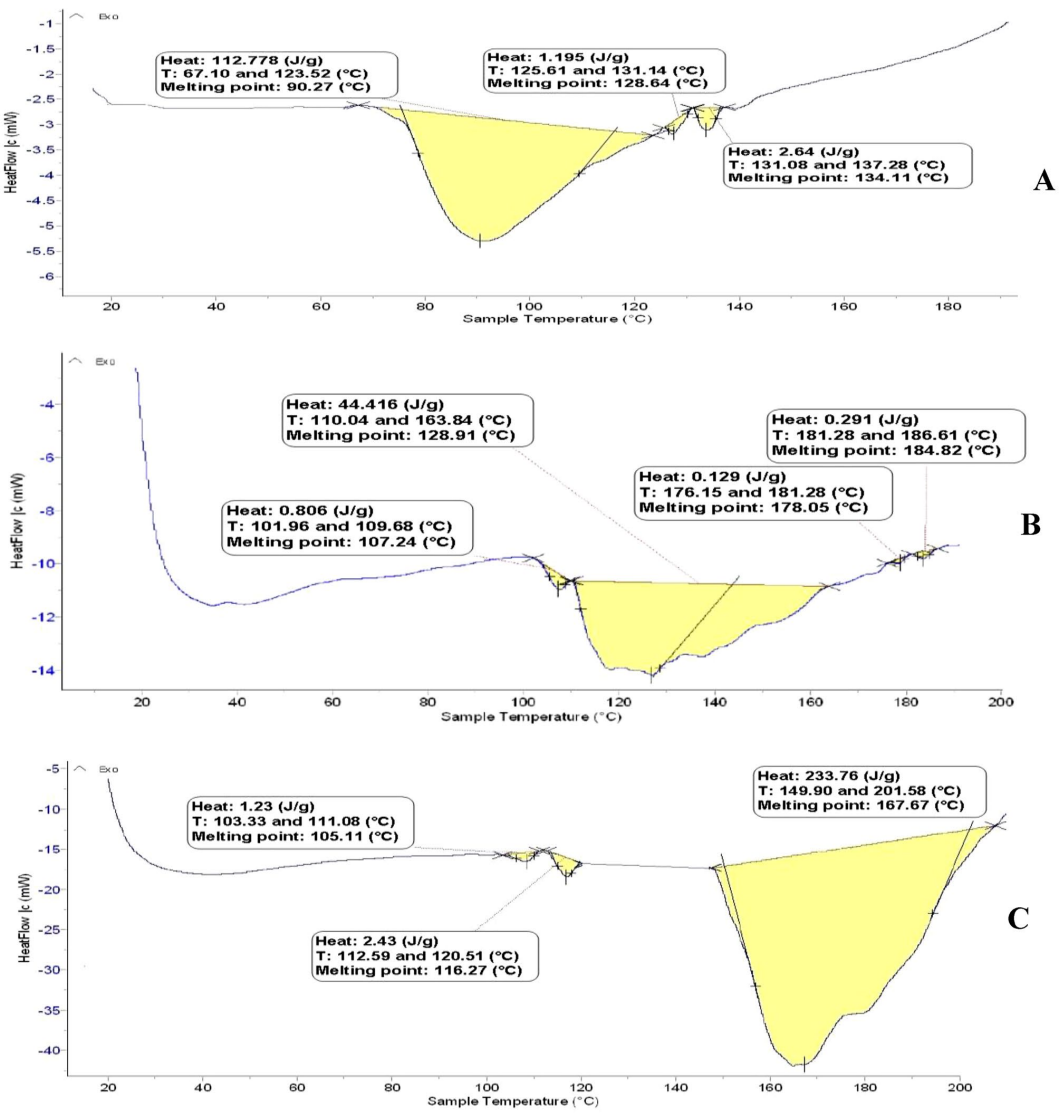
DSC profiles of PQG forms. (**A**) Propolis, (**B**) ethanol extract nano-capsules, (**C**) SCF-CO_2_ extract nano-capsules.

Three steep sorbents at temperatures representing melting points of 90.27, 128.64 and 134.11 °C are shown in the thermal schematic diagram DSC of Propolis in Fig. 5A. The extracted compounds represented a considerable area starting at 67.10 °C and ending at 123.62 °C. This sharp heat absorption peak starts from the melting point of the weak melt transition at 90.27 °C. Therefore, the extract in its free form (un-capsulated) could not be used in food applications as an additive because could not torelate high temperature^49^.

While the DSC thermogram of ethanol extract nano-capsules had four melting points in Fig. 5b (i.e. 107.24, 128.91, 178.05 and 184.82 °C), the Propolis tolerance ranged from 110.04 to 163.84 °C. It was more resistant to exposure to high temperatures than the un-capsulated Propolis sample, in which a crystalline state was not formed. This proved the success of the packaging process and the thermal stability against temperature^47^.

On the other hand, the SCF-CO_2_ nano-capsules had higher thermal stability than the other two samples. Three peaks with thermal exposure from 149.90 to 201.58 °C are shown in Fig. 5C, with a melting point of 167.67 °C. The ability of SCF-CO_2_ nano-capsules was proven by these results to be used in food applications, especially in bakery products.

#### LC–ESI–MS/MS analysis of propolis nano-capsules

**Figure 6:**
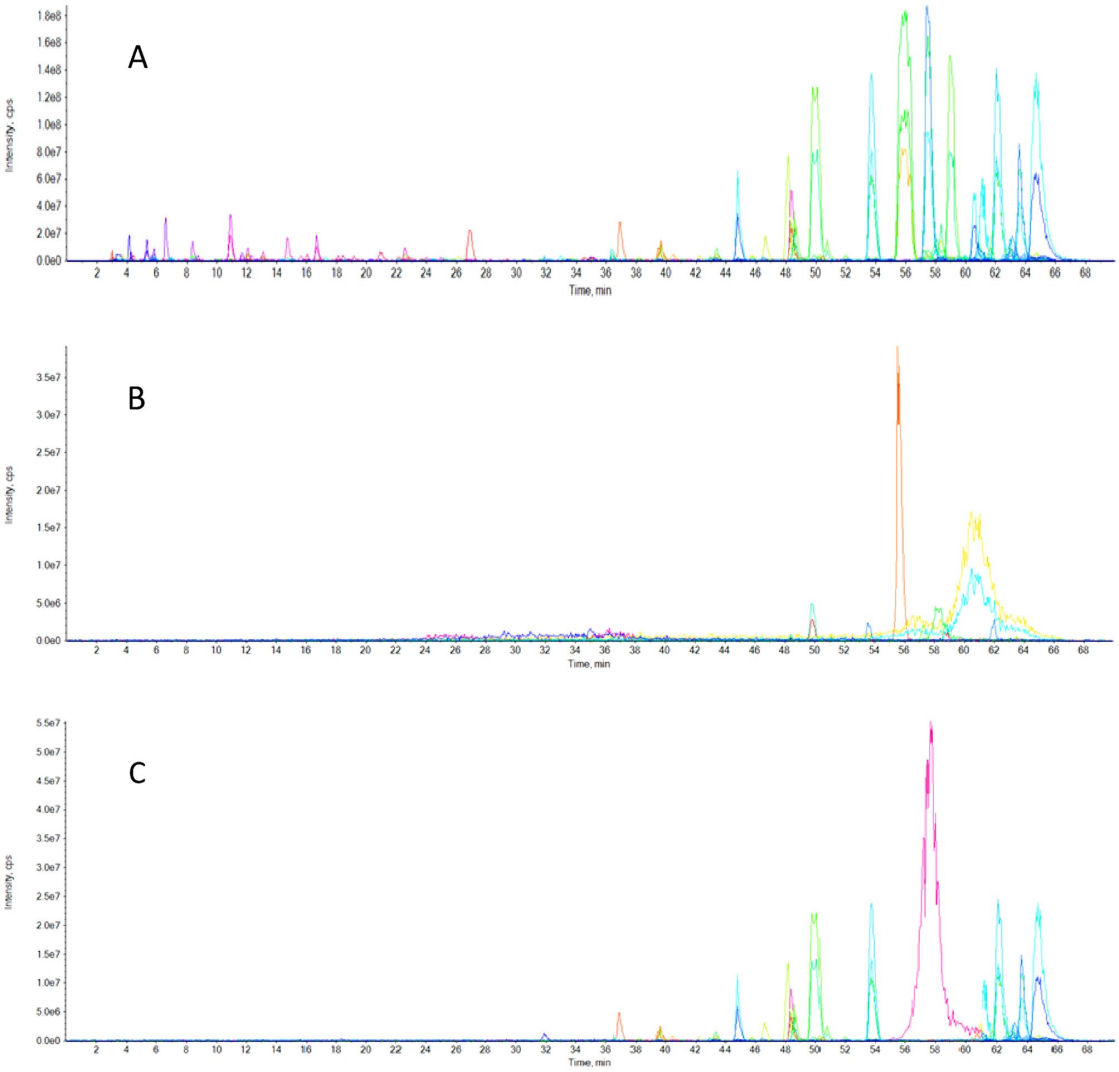
LC–ESI–MS/MS components analysis present in propolis. (**A**) Propolis, (**B**) ethanol extract nano-capsules; (**C**) SCF-CO_2_ extract nano-capsules at 50 °C.

The polyphenolic profile of different extracts could be determined using LC–ESI–MS/MS^50^. The LC–ESI–MS/MS analysis of Propolis nano-capsules of the ethanol and SCF-CO_2_ extracts at 50 °C are shown in Fig. 6A–C and Table 4. Heneicosapentaenoic acid, ketotricla-bendazole and heneicosapentaenoic acid were detected in all three propolis forms.

**Table 4:** LC–ESI–MS/MS compounds analysis of Egyptian propolis ethanol and SCF-CO_2_ extracts at 50 °C nano-capsules.

N	RT	Formula structure	Molecular weight (MW)	Putative compound name	Precursor *m/z*		
					Propolis	Nano-capsulate PQG solvent extract	Nano-capsulate PQG- SCF-CO_2_ at 50 °C extract
30	36.34	C_21_H_32_O_2_	315.091	Heneicosapentaenoic acid (NIST)	315.0914	231.0392	315.0914
31	36.88	HOOC(CH_2_)_7_COOH	187.173	Azelaic acid (NIST)	187.1725	–	187.1725
32	37.14	C_10_H_16_O_4_S	231.046	(. + −.)-Camphor-10-sulfonic acid (NIST)	–	231.0454	–
33	39.49	C_16_H_12_O_5_	283.108	Glycetein	283.1087	–	280.0259
34	39.63	C_11_H_12_O	207.160	3,4-Dimethoxycinnamic acid (NIST EL)	207.1635	–	201.2361
35	39.93	C_11_H_12_O	207.113	3,4-Dimethoxycinnamic acid (NIST EL)	207.1395	–	200.3567
36	40.49	–	245.130	Met-Pro (NIST)	245.1296	–	240.1136
37	43.40	–	271.114	No match	271.1135	–	268.1360
38	44.77	C_18_H_36_O_3_	299.108	3-Hydroxyoctadecanoic acid (NIST)	299.1035	–	291.2305
39	45.69	C_15_H_14_O_6_	287.102	3,4,2',4',6'-Pentahydroxychalcone (NIST)	287.1020	–	280.2504
40	46.49	C_22_H_16_O_4_	343.095	7-Hydroxy-3-(4-methoxyphenyl)-4-phenylcoumarin	343.0949	–	339.1055
41	46.61	C_10_H_13_N_5_O_5_	282.123	2-Hydroxyadenosine	282.1246	–	275.2158
42	48.15	C_15_H_10_O_7_	301.118	Quercetin	301.1126	–	298.2531
43	48.33	C_27_H_38_O_9_	329.115	11a-Hydroxyprogesterone	329.1154	–	321.2791
44	48.56	C_15_H_10_O_6_	285.154	Kaempferol	285.1447	–	275.1836
49	53.70	C_16_H_12_O_7_	315.101	Isorhamnetin	315.0980	271.1153	303.6513
50	55.95	C_18_H_24_O_2_	271.201	Estradiol	271.1955	242.2291	264.2894
51	56.57	C_16_H_32_O_3_	271.125	DL-.beta.-hydroxypalmitic acid (NIST)	271.1403	–	263.0258
52	57.18	C_13_H_7_C_l3_N_2_O_2_	327.121	Ketotriclabendazole	327.1222	269.0904	319.3691
53	57.89	C_15_H_10_O_5_	269.129	Genistein	269.1283	–	191.0703
54	58.07	–	242.332	Gln-Pro (NIST)	242.3310	228.2186	–
55	58.36	C_15_H_12_O_6_	287.162	Funalenone	287.1635	–	–
56	58.90	C_15_H_10_O_6_	285.151	Kaempferol	285.1507	215.3376	276.1369
60	60.64	C_18_H_36_O_3_	299.116	3-Hydroxyoctadecanoic acid (NIST)	299.1158	242.2430	–
65	61.53	C_16_H_12_O_4_	267.161	7-Hydroxy-6-methoxyisoflavone (NIST)	267.1759	–	254.1587
66	62.06	C_21_H_32_O_2_	315.151	Heneicosapentaenoic acid (NIST)	315.1539	315.0855	304.2510
67	62.55	C_21_H_32_O_2_	315.120	Heneicosapentaenoic acid (NIST)	315.0979	–	311.1054
68	62.55	C_12_H_24_O_11_	343.104	Lactitol (NIST)	343.1036	–	331.1364
69	63.08	C_11_H_12_O_3_	191.183	Ethyl *p*-coumarate (NIST)	191.1837	–	186.4542
70	63.32	C_15_H_12_O_4_	255.180	Pinocembrin (NIST)	255.1788	–	246.2408
71	63.55	C_15_H_10_O_5_	269.166	Genistein	269.1652	–	251.2208
72	63.91	C_15_H_12_O_4_	255.193	Pinocembrin (NIST)	255.2029	–	248.1344
73	64.68	C_16_H_12_O_6_	299.101	Hydroxygenkwanin	299.1002	–	282.2013
74	65.53	C_16_H_12_O_6_	299.086	Hydroxygenkwanin	299.0914	–	289.0045
76	65.59	–	284.136	No match	284.1402	–	272.0523

*RT* retention time, *SCF-CO*_*2*_ super critical fluid-carbon dioxide.

The highest concentration of flavonoids and phenolics occurred in crude propolis i.e. glycetein, quercetin, kaemferol, ethyl p-coumarate, and 3,4-dimethoxycinnamic acid were observed in both crude propolis and nano-capsules SCF-CO_2_ extracts at 50 °C. The absence of most compounds was indicated in the ethanol extract. The presence of 30, 49, 50, 52, 56 and 66 derivatives in all propolis samples in Table 4 with a similar retention time (RT) was evidenced.

The chromatograms (A, B and C) contain the peaks corresponding with the generally expected phenolic compounds were found in nano-capsules from SCF-CO_2_ at 50 °C extracts in Fig. 6. The highest component was kaempferol in the crude propolis at an RT of 56 min, and the lowest component was in the ethanol extract of PQG (285.1507 and 215.3376 *m/z*), respectively in Fig. 6.

These compounds were observed in several types of propolis sourced from different parts of the world^51^.

The biological properties of propolis were confirmed due to its high phenolic and flavonoid content, making it a food ingredient with high antioxidant activity. These data indicate that propolis may play a critical role in health beneficial effects.

#### Cytotoxic effect of human cell lines

**Figure 7:**
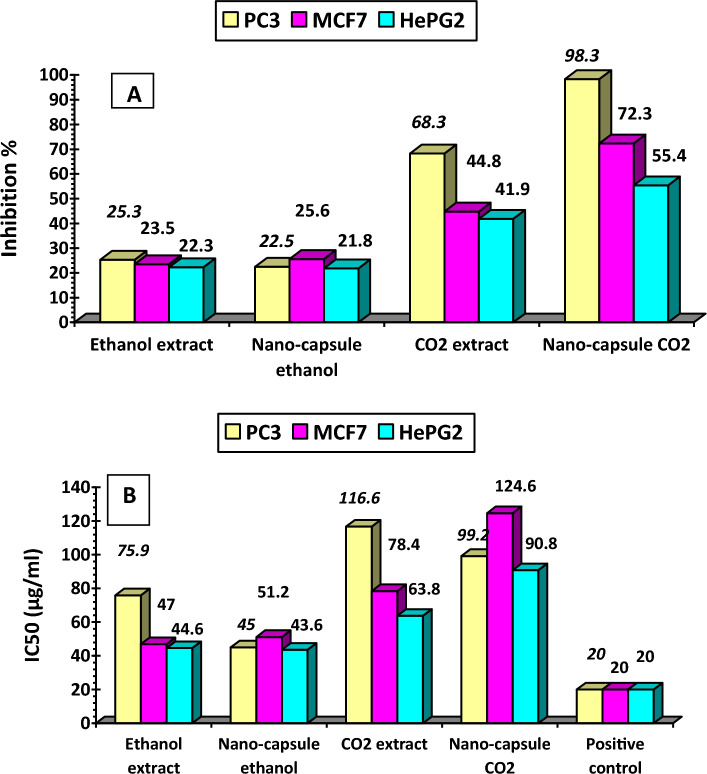
(**A,B**) Anticancer effect of propolis extracts and their nano-capsules. *IC*_*50*_ lethal concentration of the sample that causes 50% death of PC3: prostate cell line; *MCF7* human Caucasian breast adenocarcinoma, *HePG2* human hepatocellular carcinoma cell line, positive control Adriamycin (doxorubicin) after 48 h.

Egyptian propolis extracts were examined against the human tumour prostate cell line (PC3), human hepatocellular carcinoma cell line (HePG2) and human Caucasian breast adenocarcinoma (MCF7). The antitumoural effects of propolis on the three cells are documented in Fig. 7A, B.

Results in Fig. 7A showed that SCF-CO_2_ propolis extract nano-capsule increase in the inhibitory activity for the three cancer cells was 98.3, 72.3 and 55.4% for PC3, MCF7 and HePG2 respectively compared to ethanol extract and their nano-capsules being (25.3, 23.5 and 22.3%, respectively). These results indicated that there is a direct correlation between TPC concentration and cancer cell inhibition due to the presence of highest antioxidant compounds (most notably TPC and TFC)^52^.

On the other hand, IC_50_ described the lethal concentration of the sample which causes 50% death of cells in 48 h compared to the positive control (i.e. Doxorubicin, an anticancer drug for hematologic and solid tumors, was used as the positive control).

Results in Fig. 7B showed that the lower values belonged to SCF-CO_2_ propolis nano-capsule in the case of HePG2 (90.8 µg/mL), PC3 (99.2 µg/mL), and MCF7 (124.6 µg/mL) followed by SCF-CO_2_ extract for HePG2 cells (63.8 µg/mL), MCF7 cells (78.4 µg/mL) and PC3 cells (116.6 mg/mL).

While IC_50_ for ethanol extract was (44.6 µg/mL) for PC3 cells followed by (47 g/mL) and (75.9 µg/mL) for MCF7cells and PC3 respectively .The lower was for ethanol nano-capsule extract in all cells.

The recommendation of NCI stated that IC_50_ of crude extract, if incubated from 48 to 72 h, has cytotoxic activity in vitro less than 20 and 4 µg/ml pure compounds^53^.

Different types of honey and propolis extracts have been indicated in many reports to significantly inhibit cell growth and reduce the differentiation or proliferation of cells from various tumour cell lines^54^. Cancer cell inhibition should be evaluated to develop new anticancer drugs. Based on these criteria, nano-capsule CO_2_ propolis extracts were assessed for their inhibition as candidate anticancer cancer drugs for human hepatocellular, prostate and human Caucasian breast adenocarcinoma.

### Application

#### Sensory evaluation of crackers fortified with propolis extracts

**Figure 8:**
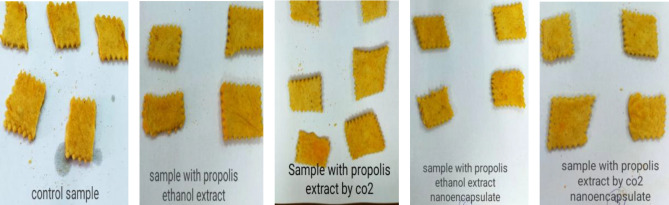
Crackers samples unfortified and fortified with different types of propolis extracts and nano-capsules.

The sensory scores of crackers fortified with propolis extracts and their nano-capsules are shown in Table 5 and Fig. 8.

**Table 5:** Sensory evaluations of fortified crackers with different types of Propolis extracts and nano-capsules.

Cracker samples	Appearance (10)	Color (15)	Thickness (15)	Crispness (15)	Shrinkage (15)	Taste (15)	Odor (15)	Total overall acceptability (100)
Control	9.62 ± 0.5^a^	14.31 ± 0.4^ab^	10.63 ± 0.4^a^	12.85.25 ± 0.3^a^	12.95 ± 0.4^a^	13.23 ± 0.5^ab^	13.25 ± 0.3^ab^	86.84
FP-EE	8.35 ± 0.5^ab^	12.03 ± 0.5^b^	10.42 ± 0.5^ab^	11.56 ± 0.4^ab^	11.31 ± 0.5^b^	12.55 ± 0.6^b^	11.11 ± 0.4^b^	77.33
FP-SCF	8.94 ± 0.5^ab^	12.27 ± 0.5^b^	10.68 ± 0.5^a^	11.62 ± 0.4^ab^	11.79 ± 0.5^b^	12.76 ± 0.5^b^	12.03 ± 0.4^b^	80.09
FP-EEN	9.35 ± 0.6^b^	13.35 ± 0.6^bc^	11.38 ± 0.5^b^	13.78 ± 0.6^b^	12.52 ± 0.6^bc^	13.09 ± 0.6^b^	12.82 ± 0.5^c^	86.29
FP-SCFN	9.71 ± 0.7^b^	13.40 ± 0.6^bc^	11.35 ± 0.6^bc^	13.04 ± 0.7^bc^	12.88 ± 0.6^c^	13.11 ± 0.6^bc^	13.08 ± 0.5^cd^	86.57

*FP-EE* cracker fortified with PQG ethanol extract, *FP-SCF* cracker fortified with propolis SCF-SO_2_ extract at 50 °C, *FP-EEN* cracker fortified with propolis ethanol extract nano-capsules, *FP-SCFN* cracker fortified with propolis SCF-SO_2_ extract at 50 °C nano-capsules.

Different superscripts in the same column or row are significant differences at P ≤ 0.05 level.

The control sample was characterized by the highest (p ˂ 0.05) total overall acceptability (86.84%) among the cracker samples tested (Table 5). At the same time, the lower scores of crackers fortified with Propolis ethanol extract than the two nano-capsules sample may be due to the gum taste of the propolis extract. The overall acceptability of crackers fortified with Propolis SCF-CO_2_ at 50 °C and ethanol extracts nano-capsules was 86.57 and 86.29, respectively.

These crackers (FE-EEN and FP-SCFN) obtained the highest score, probably because they acquired the fragility and porosity properties of the sodium alginate capsules and their ability to bind water and reduce its content^55^. For a long time, propolis bioactive ingredients have been used in many applications. Many new functional food products have appeared in the markets in recent years to respond to health problems faced by consumers.

#### Storage stability of fortified cracker samples

##### Antioxidant activity of stored crackers

The DPPH scavenging activity of the stored cracker samples fortified with propolis extracts and their nano-capsules were measured compared with the control samples during the storage period of 90 days, as shown in Table 6.

**Table 6:** Storage stability of cracker samples fortified with various propolis extracts.

Fortified cracker samples	DPPH radical scavenging activity %				
	Propolis extract (0.6 g) before cracker fortification	Zero time after baking	After 30 days storage	After 60 days storage	After 90 days storage
FP-EE	76.73 ± 2.8^ab^	54.58 ± 1.9^a^	48.33 ± 1.9^a^	42.51 ± 2.1^a^	36.18 ± 1.6^a^
FP-SCF	96.54 ± 3.1^c^	76.84 ± 3.1^ab^	68.13 ± 2.5^b^	59.42 ± 2.3^b^	48.33 ± 2.1^ab^
FP-EEN	64.88 ± 2.6^a^	62.43 ± 2.3^b^	60.92 ± 2.2^b^	58.14 ± 2.2^b^	55.07 ± 2.3^b^
FP-SCFN	92.57 ± 2.9^d^	90.72 ± 3.1^c^	88.27 ± 2.8^c^	86.24 ± 2.7^c^	84.79 ± 2.7^c^

*FP-EE* cracker fortified with propolis ethanol extract, *FP-SCF* cracker fortified with propolis SCF-SO_2_ extract at 50 °C, *FP-EEN* cracker fortified with propolis ethanol extract nano-capsules, *FP-SCFN* cracker fortified with propolis SCF-SO_2_ extract at 50 °C nano-capsules.

Different superscripts in the same column or row are significant differences at P ≤ 0.05 level.

A clear and noticeable significant decrease in DPPH (p ˂ 0.05) between the fortified crackers with ethanol and SCF-CO_2_ at 50 °C extracts with increasing storage time are shown in Table 6. From zero to 90 days of storage, the decrease was between 54.58 and 36.18% for the ethanol extract and 76.84% and 48.33% for the SCF-CO_2_ extract. This was due to the high sensitivity of the un-capsulated extract to storage period. The cracker samples fortified with nano-capsules of ethanol and SCF-CO_2_ at 50 °C extracts had slight decreases, from 62.43 to 55.07% and from 90.72 to 84.79% after 90 days storage respectively, due to the ability of encapsulation to protect the extract from exposure to high temperatures.

These results were indications that crackers fortified with nano-capsulate propolis SCF-CO_2_ extract at 50 °C had a higher ability to retain antioxidant activity during the storage period, followed by the crackers fortified with propolis nano-capsulate ethanol extract. The encapsulation process was able to store the propolis extract for nine months in solid-state form, as mentioned by Ticiano et al*.*^56^.

##### Peroxide value (PV) of the crackers

The changes in PV of the five stored cracker samples that occurred during the storage period are shown in Table 7. The increase of peroxide values was relatively low in the cracker fortified with PQG ethanol and SCF-CO_2_ at 50 °C extracts nano-capsules, being 0.11 and 0.08, respectively. This result occurred because of the ability of capsules to prevent the oxidation of fat and rancidity, increasing the shelf life of crackers.

**Table 7:** Peroxide values (PV) of cracker samples during incubation period (days).

Days	Peroxide value				
	Control	FP-EE	FP-SCF	FP-EEN	FP-SCFN
0	0.13 ± 0.01^a^	0.13 ± 0.01^a^	0.13 ± 0.01^a^	0.13 ± 0.01^a^	0.13 ± 0.01^a^
30	0.75 ± 0.04^b^	0.62 ± 0.03^b^	0.59 ± 0.02^b^	0.18 ± 0.01^ab^	0.16 ± 0.02^ab^
60	0.96 ± 0.06^bc^	0.71 ± 0.04^b^	0.64 ± 0.03^b^	0.21 ± 0.02^ab^	0.19 ± 0.02^ab^
90	1.23 ± 0.08^cd^	0.84 ± 0.06^c^	0.76 ± 0.03^bc^	0.24 ± 0.02^b^	0.21 ± 0.02^b^
Increase T0–T90	1.1	0.71	0.63	0.11	0.08

*FP-EE* cracker fortified with PQG ethanol extract, *FP-SCF* cracker fortified with SCF-SO_2_ at 50 °C extract from PQG, *FP-EEN* cracker fortified with PQG ethanol extract nano-capsules, *FP-SCFN* cracker fortified with PQG-SCF-SO_2_ at 50 °C extract nano-capsules.

Different superscripts in the same column or row are significant differences at P ≤ 0.05 level.

## Conclusions and prospects

Currently, there is a significant interest directed towards the bioactive compounds of plants to build up a robust immune system. This future vision led researchers to discover a novel technique for extracting these bioactive compounds facilitate and increase the yield, improving the process without degradation. The functional food market has expanded over the years due to the demand of consumers for healthy food products, reducing the use of synthetic preservatives and antioxidants and improving the quality of products^57^.

Nano-capsulation technologies in the food industry, mainly nano-structured food ingredients, improve solubility, sensory properties and stability during storage^58,59^. Controlling food safety and risk assessments of novel materials added to food encourage many countries to establish powerful platforms^60^. However, the toxicological repercussions of these new materials and ethical issues are still limited and remain to be tested.

We choose nano-capsules of ethanolic and SCF-CO_2_ extracts at 50 °C in this study. These nanocapsules were characterized by the high extraction yields and supercritical extracts, which provided higher flavonoid concentrations than EEP. Such a result indicates essential compounds in the fractionation of propolis compounds, as made by several authors before being commercialised. Propolis nano-capsules (SCF-CO_2_ at 50 °C extracts) had robust inhibitory effects on human tumour growth, potentially preventing oxidative damage and inducing apoptosis and immune stimulation. The crackers fortified with these two propolis extract nano-forms proved their overall acceptability, improved their stability and extended their shelf life. This product could be considered a new functional food that the food industry could process to improve human health. Finally, numerous futures expand for nano-capsulation in the food industry will enhance the standard of living of people.

## Data Availability

Tip 1: In general, different methods were used to extract the bioactive ingredients in propolis, and the best extraction method was to use supercritical fluid carbon dioxide as shown in Table 1. The data issued in Table 2 also shows the content of phenols and total flavonoids for those extracts, and Figs. 1 and 2 show the antioxidant activity of those extracts through reducing power and DPPH, and Fig. 3 confirms the total antioxidant activity of the extracts. And the comparison between them (free and encapsulated extracts in nano-capsules). Table 3 shows the antioxidant activity of the extracts before and after nano-encapsulation, which showed significant differences for the encapsulated extracts higher than the un-encapsulated ones. The physical and chemical properties of those free and encapsulated extracts were studied, and the encapsulation efficiency was studied. Figure 4 shows the morphological composition of the particles before and after nano-encapsulation. Figure 5 shows the thermal stability of the extracts before and after nano-encapsulation to determine the extent to which they can be applied in the field of foods that are exposed to high temperatures. The toxicity of these extracts on cancer cells of the liver and prostate was also studied, and their effects on cell vitality and inhibition of cancer cells were studied. Finally, the applied study was done by adding it to crackers (bakery products) by comparing the alcoholic extract and the extract with CO_2_ before and after nano-encapsulation, and studying the extent of storage stability for those products that contain natural antioxidants, which were favored by consumers. Tip 2: Data is "on demand" and all data in a study is available to scholars in the same field. Tip 3: All data we create is disclosed. "All the data mentioned in this paper are available and help in understanding the importance of the study."
